# Stability of BDNF in Human Samples Stored Up to 6 Months and Correlations of Serum and EDTA-Plasma Concentrations

**DOI:** 10.3390/ijms18061189

**Published:** 2017-06-03

**Authors:** Maryna Polyakova, Haiko Schlögl, Julia Sacher, Maren Schmidt-Kassow, Jochen Kaiser, Michael Stumvoll, Jürgen Kratzsch, Matthias L. Schroeter

**Affiliations:** 1 Department of Neurology, Max Planck Institute for Human Cognitive and Brain Sciences, 04103 Leipzig, Germany; sacher@cbs.mpg.de (J.S.); schroet@cbs.mpg.de (M.L.S.); 2 Clinic for Psychiatry and Psychotherapy, University of Leipzig, 04103 Leipzig, Germany; 3 LIFE—Leipzig Research Center for Civilization Diseases, University of Leipzig, 04103 Leipzig, Germany; juergen.kratzsch@medizin.uni-leipzig.de; 4 Department of Endocrinology, University Hospital Leipzig, 04103 Leipzig, Germany; michael.stumvoll@medizin.uni-leipzig.de; 5 Institute of Medical Psychology, Goethe University Frankfurt, 60528 Frankfurt, Germany; schmidt-kassow@med.uni-frankfurt.de (M.S.-K.); j.kaiser@med.uni-frankfurt.de (J.K.); 6 Institute of Laboratory Medicine, Clinical Chemistry and Molecular Diagnostics, University Hospital Leipzig, 04103 Leipzig, Germany; 7 Clinic for Cognitive Neurology, University of Leipzig, 04103 Leipzig, Germany

**Keywords:** BDNF, brain-derived neurotrophic factor, serum, EDTA-plasma, stability, inter-assay precision, acceptable change limit

## Abstract

Brain-derived neurotrophic factor (BDNF), an important neural growth factor, has gained growing interest in neuroscience, but many influencing physiological and analytical aspects still remain unclear. In this study we assessed the impact of storage time at room temperature, repeated freeze/thaw cycles, and storage at −80 °C up to 6 months on serum and ethylenediaminetetraacetic acid (EDTA)-plasma BDNF. Furthermore, we assessed correlations of serum and plasma BDNF concentrations in two independent sets of samples. Coefficients of variations (CVs) for serum BDNF concentrations were significantly lower than CVs of plasma concentrations (*n* = 245, *p* = 0.006). Mean serum and plasma concentrations at all analyzed time points remained within the acceptable change limit of the inter-assay precision as declared by the manufacturer. Serum and plasma BDNF concentrations correlated positively in both sets of samples and at all analyzed time points of the stability assessment (*r* = 0.455 to *r*_s_ = 0.596; *p* < 0.004). In summary, when considering the acceptable change limit, BDNF was stable in serum and in EDTA-plasma up to 6 months. Due to a higher reliability, we suggest favoring serum over EDTA-plasma for future experiments assessing peripheral BDNF concentrations.

## 1. Introduction

Brain-derived neurotrophic factor (BDNF) is one of the best studied neural growth factors. Recently, BDNF became a candidate biomarker for mood disorders and schizophrenia [1,2]. Nevertheless, studies investigating BDNF lack reproducibility [3]. Confounders, which are often not taken into account, include the pre-analytical treatment of samples [4] and reproducibility of the analytical method [5].

Currently, at least two distinct blood compartments for BDNF are known: (1) circulating BDNF in plasma, and (2) the platelet pool of BDNF. The latter is reflected by serum concentrations because BDNF is secreted into serum by activated platelets during blood clotting. Concentrations in serum are typically higher than concentrations in plasma [6]. Analysis of BDNF in both compartments may be of interest, because plasma BDNF concentrations reflect acute changes in humoral BDNF, and serum concentrations reflect the platelet pool of BDNF [2].

Relationships between serum and plasma BDNF are also controversially discussed. High positive correlations between serum and plasma BDNF concentrations in healthy volunteers have been reported [7]. However, other studies could not reproduce these findings [4,8]. Physical exercise, which was found to increase BDNF concentrations, influenced BDNF concentrations in serum to a higher degree than in plasma [9]. Contrarily, in another study the effect of exercise was larger in plasma samples than in serum samples [10]. Serum BDNF increased after longitudinal aerobic exercise [11,12] and showed an association with thyroid hormone concentrations during antidepressant treatment [13], while plasma BDNF did not [11,13]. Even opposite effects of interventions on BDNF concentrations in serum and plasma have been reported: surgical stress increased plasma BDNF but decreased serum BDNF [14].

Plasma BDNF concentrations are strongly influenced by pre-analytical conditions. They increase up to seven-fold with increasing pre-centrifugation delay [4]. Serum BDNF concentrations increase in the first 30 min after blood draw while clotting occurs, but after that seem to be stable [4,15,16,17]. In clinical studies, blood samples are often deep frozen and stored before analysis, which may be a further factor influencing the concentrations. In an observational study a negative correlation of serum BDNF with storage at −80 °C was reported [18].

In this study, we addressed two questions. First, we assessed the stability of serum and ethylenediaminetetraacetic acid (EDTA)-plasma BDNF when stored at room temperature, at −80 °C, and after repeated freeze/thaw cycles. We hypothesized that serum and EDTA-plasma BDNF concentrations would remain inside the acceptable change limit [19] as calculated using the inter-assay precision reported by the manufacturer of the BDNF analysis kit [20]. Second, we assessed the correlation between serum and plasma BDNF in two independent sets of samples. Based on previous data, we hypothesized a positive, albeit weak, correlation. Finally, we compared the reliability between serum and plasma BDNF by calculating the coefficients of variation.

## 2. Results

### 2.1. Baseline Data

**Figure 1:**
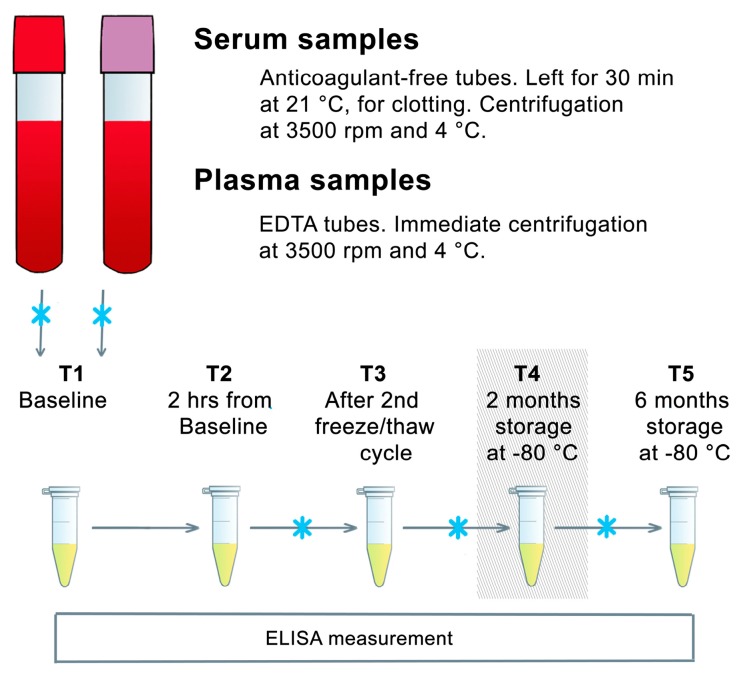
Schematic design of the stability study. T1 to T5 represent time points for BDNF measurements. Blue crosses signify freezing at −80 °C. The grey box at T4 indicates the time point which was excluded from the analysis due to a measurement error. BDNF: brain-derived neurotrophic factor, EDTA: ethylenediaminetetraacetic acid, ELISA: enzyme-linked immunosorbent assay, hrs: hours, min: minutes, rpm: rounds per minute, T: time point.

The study design is depicted in Figure 1. Anthropometric data of participants and measured BDNF concentrations are presented in Table 1. In group one, mean BDNF concentrations at baseline were 19.9 ± 0.8 ng/mL (mean ± SEM; SD 5.6; range 11.8–34.4) for serum and 3.2 ± 0.2 ng/mL (1.5; 0.8–6.7) for EDTA-plasma samples. In group two, mean BDNF concentrations were 24.3 ± 1.3 ng/mL (5.9; 16.1–40.8) for serum and 5.1 ± 0.8 ng/mL (3.5; 0.3–18.0) for EDTA-plasma samples.

### 2.2. Stability Tests

**Figure 2:**
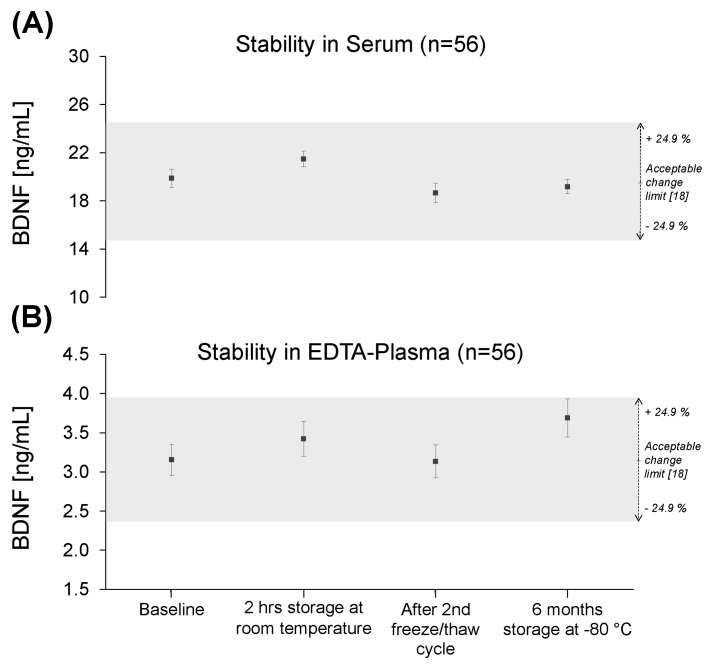
BDNF concentrations at “Baseline” (T1), “2 hrs storage at room temperature” (T2), “After 2nd freeze/thaw cycle” (T3), and “6 months storage at −80 °C” (T5). (**A**) Serum samples, (**B**) EDTA-plasma samples. Grey box: 24.9% acceptable change limit as calculated with the inter-assay precision declared by the manufacturer of the BDNF ELISA kit [20]. All error bars are SEM. BDNF: brain-derived neurotrophic factor, EDTA: ethylenediaminetetraacetic acid, ELISA: enzyme-linked immunosorbent assay, hrs: hours, SEM: standard error of the mean.

**Figure 3:**
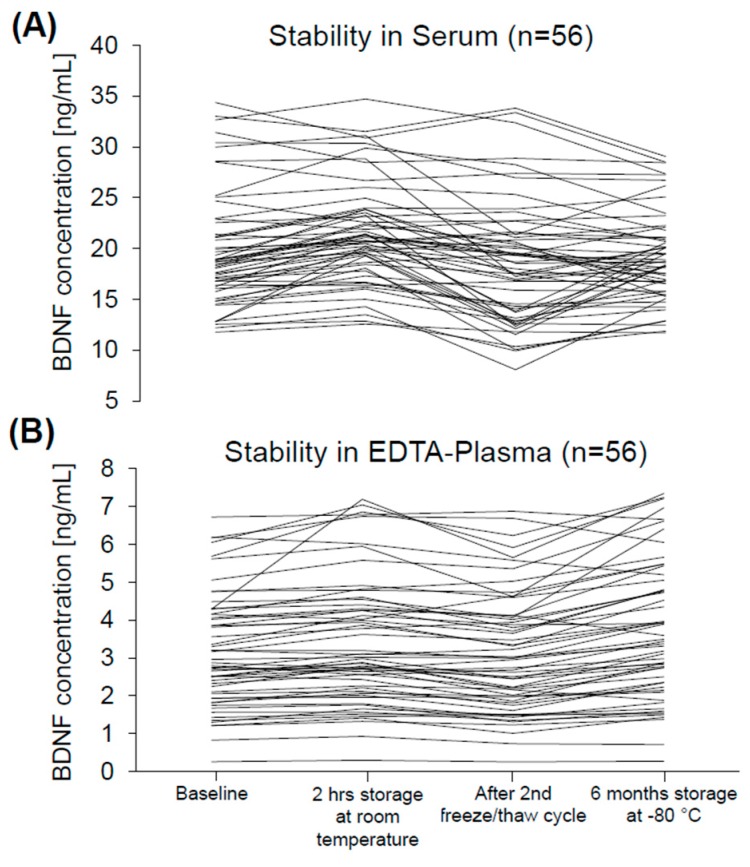
Individual changes in serum (**A**) and EDTA-plasma (**B**) BDNF at “Baseline” (T1), “2 hrs storage at room temperature” (T2), “After 2nd freeze/thaw cycle” (T3), and “6 months storage at −80 °C” (T5). BDNF: brain-derived neurotrophic factor, EDTA: ethylenediaminetetraacetic acid, hrs: hours.

In group one, mean serum BDNF concentrations at time point two (T2, “2 hrs storage at room temperature”) were 21.5 ± 0.7 ng/mL (4.9; 11.8–34.4), at time point three (T3, “After 2nd freeze/thaw cycle”) were 18.7 ± 0.8 ng/mL (6.0; 8.1–33.8), and at time point five (T5, “6 months storage at −80 °C”) were 19.2 ± 0.6 ng/mL (4.4; 11.7–29.1). Mean EDTA-plasma BDNF concentrations at T2 were 3.4 ± 0.2 ng/mL (1.7; 0.3–7.2), at T3 were 3.1 ± 0.2 ng/mL (1.6; 0.2–6.9), and at T5 were 3.7 ± 0.2 ng/mL (1.8; 0.3–7.3) (Table 1, Figure 2 and Figure 3). The coefficients of variation (CVs) at all analyzed time points were significantly smaller in serum than in plasma (*n* = 245, *t* = −5.4, *d*_f_ = 4, *p* = 0.006, Table 1).

One serum BDNF value at measurement T3 was identified as an outlier using the outlier labeling rule proposed by Hoaglin et al. [21], and one plasma BDNF value at T5 was identified as an outlier, because the value exceeded the upper limit of detection (8 ng/mL) of the used BDNF enzyme-linked immunosorbent assay (ELISA) kit. Both samples were excluded from the respective analyses. The data of one sample (both serum and plasma) at T5 was missing, most likely due to a data transfer error. All time points of the respective sample were excluded from the stability analysis. This resulted in a total sample size for the stability tests of *n* = 56 for serum and plasma. At time point four (T4, “2 months storage at −80 °C”) six plasma values were above the upper limit of the measurement range. Also all other measurements at this time point showed very large variations. After looking at the data in detail (Appendix A and Appendix B) we assumed an unknown measurement error and excluded the whole time point from any further analyses.

Mean BDNF concentrations at all included time points (T2, T3, T5) remained within the acceptable change limit calculated with the inter-assay precision reported by the manufacturer of the ELISA kit [20] (Figure 2). Repeated measures of analysis of variance (ANOVA) and further post-hoc analyses between single time points would not be interpretable and therefore were not performed.

### 2.3. Correlations between Serum and EDTA-Plasma BDNF

**Figure 4:**
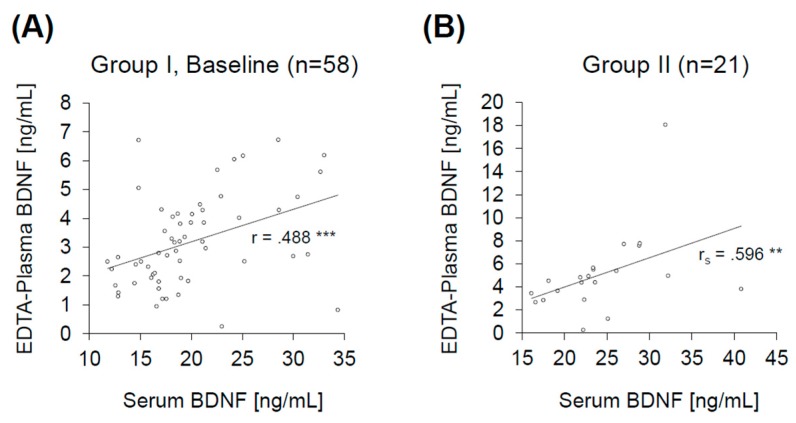
Correlations of serum and EDTA-plasma BDNF in Group I (“Baseline”, T1) (**A**) and Group II (**B**). BDNF: brain-derived neurotrophic factor; EDTA: ethylenediaminetetraacetic acid. ** *p* < 0.01, *** *p* < 0.001.

**Figure A3:**
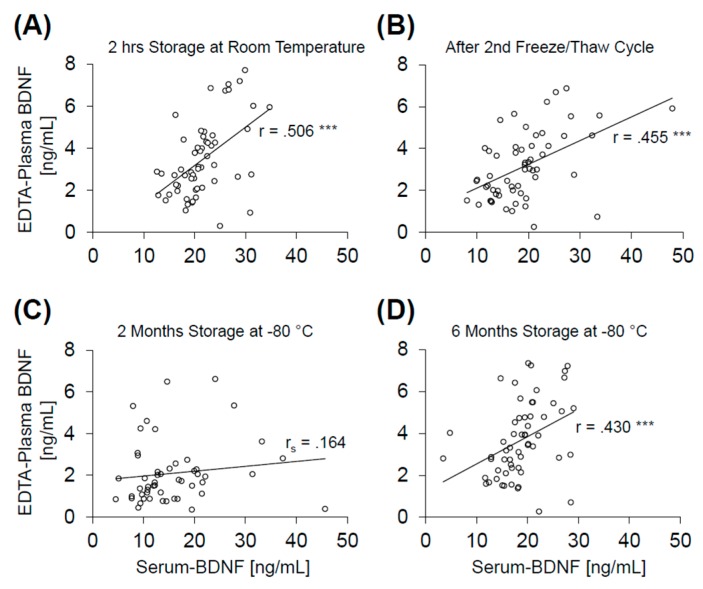
Correlations of serum and EDTA-plasma BDNF concentrations for time points (**A**) “2 hrs storage at room temperature” (T2), (**B**) “After 2nd freeze/thaw cycle” (T3), (**C**) “2 months storage at −80 °C” (T4), (**D**) “6 months storage at −80 °C” (T5). BDNF: brain-derived neurotrophic factor, EDTA: ethylenediaminetetraacetic acid, hrs: hours.

Serum and EDTA-plasma BDNF concentrations correlated positively in both groups of samples (Figure 4). This positive correlation remained significant for all time points included in the analysis (*r* = 0.455 to *r*_s_ = 0.596; *p* < 0.004) (Figure 4 and Figure A3).

## 3. Discussion

Our study compared BDNF concentrations in serum and EDTA-plasma and their stability over time. CVs for serum BDNF values were significantly lower than for EDTA-plasma values in all comparisons. Mean serum and EDTA-plasma BDNF concentrations at all time points remained within the acceptable change limit for inter-assay precision.

Our findings of smaller CVs for serum as compared to EDTA-plasma are consistent with previous findings [4,15,16] and are explained by the complex dynamics of BDNF in peripheral blood. The majority of serum BDNF is released from platelets during the clotting process [6]. In plasma, measured BDNF concentrations are considered to represent humoral BDNF and are much lower than in serum [22]. Therefore, plasma measurements are highly sensitive to BDNF released from platelets [23]. Although EDTA in tubes for plasma preparation prevents coagulation, it does not completely stop platelet degranulation. It has been shown that platelets continue to degranulate in the presence of anticoagulants [24]. This leads to an increase in BDNF concentrations in plasma with increasing pre-centrifugation delay [15]. Small pre-centrifugation delays, often unavoidable in clinical studies, may have occurred. This may have led to varying increases in BDNF in our plasma samples, and thus higher CVs than in serum. To conclude, our results confirm previous data in two independent samples. We support recommendations to prefer serum over EDTA-plasma when determining concentrations of BDNF in clinical studies. For the reliable assessment of short term changes (i.e., changes occurring in the scale of minutes) in BDNF concentrations, further studies are needed to establish pre-analytical protocols of plasma BDNF sample preparation.

We observed a positive correlation between serum and EDTA-plasma BDNF concentrations in two independent sets of samples, both consisting of healthy volunteers. These results confirm the positive correlation reported previously [7]. Since BDNF concentrations differ depending on the anticoagulant used [4], we suggest a specification of the term “plasma BDNF” to the anticoagulant used, in our case “EDTA-plasma BDNF”.

In our analysis of the stability of BDNF, both mean serum and EDTA-plasma BDNF concentrations at “Baseline” (T1), “2 hrs storage at room temperature” (T2), “After 2nd freeze/thaw cycle” (T3), and “6 months storage at −80 °C” (T5) remained inside the acceptable change limit of the R&D assay used. Thus, with our method of analysis we cannot prove any influence of these storage conditions or of repeated freeze/thawing on BDNF concentrations. The inter-assay variation of our ELISA kit was 9% (mean for all concentration ranges) [20], resulting in an acceptable change limit of ±24.9%. All samples at one time point were measured with the same assay, and for every time point one assay was used. A statistical analysis using ANOVA and further post hoc analyses would not account for this inter-assay variability and thus would not be interpretable. Changes in BDNF concentrations smaller than the acceptable change limit would be undetectable.

Several limitations of our study need to be discussed. First, blood from participants was not collected on the same day, but within one month, leading to differences in storage time at −80 °C before baseline analysis. However, such a logistic delay is typical for clinical studies and cannot always be excluded, and our results demonstrate that storage up to six months at −80 °C does not affect BDNF concentrations. Second, samples of group two were only given 20 min for coagulation. A study, which was published after we performed our analyses, reported an increase of serum BDNF concentrations with time when comparing coagulation times of 10 vs. 30 min [17]. A comparison of 20 and 30 min coagulation time has not yet been performed to our knowledge. We cannot exclude that the serum BDNF values would be slightly higher if 30 min instead of 20 min coagulation time had been allowed for the serum samples of group two as well. Third, the ELISA kit we used did not show the best performance of six tested ELISA kits in a previous study [5]. Our kits were purchased prior to the publication of Polacchini et al.’s research, so we could not implement these recent results into our protocol. Fourth, our sample size was smaller than in other studies assessing correlations of serum and plasma BDNF concentrations [7], but substantially larger than in previous stability studies [4,15,16]. Fifth, the measurement at the “2 months storage at −80 °C” time point (T4) likely resulted from a laboratory error, but we cannot prove which error led to the data.

## 4. Materials and Methods

### 4.1. Participants

Blood draws were performed in 2010, and laboratory analyses were performed in 2010 and 2011. Two independent groups of participants were recruited. Group one consisted of 15 healthy volunteers. Blood was drawn twice from each volunteer, 106–180 min apart, on two different days, respectively. One volunteer missed both blood draws on one of the two study days due to a cold, resulting in a total sample size of *n* = 58 samples. Blood draws were conducted within one month. To avoid influences of sex, and to exclude influences of the female hormonal cycle, group one included only male volunteers. Group two consisted of 21 healthy volunteers (nine female). From each volunteer of this group, blood was drawn once. All participants gave informed consent for participation in the study. The research protocol was approved by the ethics committee of the University of Leipzig (approval No. 246-2009-01122009 from 01.12.2009) and was in accordance with the latest version of the Declaration of Helsinki.

### 4.2. Pre-Analytical Blood Sample Processing

Blood was drawn by venipuncture. For serum samples, 7.5 mL tubes were used (Sarstedt, Nümbrecht, Germany), and after blood draw these were stored at room temperature (approx. 21 °C) for 30 (group one) or 20 (group two) min to allow for complete coagulation. Then, the tubes were centrifuged for 10 min at 3500 rpm at 4 °C, then pipetted into 1 mL aliquots in plastic vials (2 mL volume size, Eppendorf, Hamburg, Germany) and deep frozen at −80 °C until further analysis. For plasma samples, 9 mL EDTA-tubes were used (Sarstedt). After blood draw, the EDTA-tubes were immediately centrifuged (same parameters as above) and equally stored in 1 mL aliquots at −80 °C.

### 4.3. BDNF Analysis

The serum and EDTA-plasma samples of group one were analyzed at five different time points (Figure 1). The sample was taken out of the −80 °C freezer and 120 min were allowed for thawing at room temperature (21 °C) before analysis (“Baseline”; T1). After analysis, the same aliquot was stored for two hours at room temperature and then analyzed again (“2 hrs storage at room temperature”; T2) to test the short term stability of BDNF at room temperature. Subsequently, this aliquot was deep frozen, thawed again the next day, and analyzed to test the stability of BDNF after a repeated freeze/thaw cycle (“After 2nd freeze/thaw cycle”; T3). Then, this aliquot was again deep frozen and stored at −80 °C for two months before the next analysis (“2 months storage at −80 °C”; T4). After that, this aliquot was again deep frozen and stored at −80 °C for another four months before the last analysis (“6 months storage at −80 °C”; T5). BDNF was measured using the Quantikine human BDNF Immunoassay from R&D Systems, Inc., Minneapolis, MN, USA following manufacturer’s instructions [20].

### 4.4. Statistical Tests

Statistical analyses were performed using SPSS version 22 (IBM, Chicago, IL, USA). Data distributions were assessed before the analyses. To control for the influence of outliers, we used the outlier labeling rule proposed by Hoaglin et al. [21]. CVs were calculated as the ratio of SD to the mean. Statistical differences between serum and EDTA-plasma CVs were assessed using paired sample Student’s *t*-tests. The two-tailed α-level was set to 0.05. The mean inter-assay precision for all three measurement ranges as declared by the manufacturer [20] was multiplied by 2.77 to obtain the acceptable change limit [19]. To evaluate any potential relationship between serum and EDTA-plasma BDNF concentrations, we calculated Pearson’s or Spearmen’s coefficients of correlation, based on data distributions. For all analyses, means, SEMs, SDs, and ranges are presented.

## 5. Conclusions

When considering the acceptable change limit of the used BDNF assay, concentrations in both serum and EDTA-plasma where stable after 2 hrs storage at room temperature, after a repeated freeze/thaw cycle, and after 6 months storage at −80 °C. Serum and EDTA-plasma BDNF concentrations were positively correlated. Serum measurements showed lower variability than EDTA-plasma measurements, confirming previous studies. Therefore, we suggest favoring serum over EDTA-plasma for future experiments assessing peripheral BDNF concentrations.

## Figures and Tables

**Table 1:** Anthropometric data of participants and respective BDNF concentrations in serum and EDTA-plasma.

Measurements	Number of Participants (Male)	Number of Samples	Age (Years) ± SD	BMI (kg/m²) ± SD	Serum BDNF		EDTA-Plasma BDNF	
					ng/mL ± SD	CV	ng/mL ± SD	CV
*Group I*								
Baseline (T1)	15 (15)	56	25.2 ± 2.9	24.6 ± 4.7	19.9 ± 5.6	28%	3.2 ± 1.5	47%
2 hrs storage at RT (T2)					21.5 ± 4.9	23%	3.4 ± 1.7	49%
2nd freeze/thaw cycle (T3)					18.7 ± 6.0	32%	3.1 ± 1.6	50%
6 months storage at −80 °C (T5)					19.2 ± 4.4	23%	3.7 ± 1.8	49%
*Group II*								
Baseline	21 (9)	21	38.4 ± 11.8	22.9 ± 3.4	24.3 ± 5.9	24%	5.1 ± 3.5	70%

Mean ± standard deviation (SD). BDNF: brain-derived neurotrophic factor, BMI: body mass index, CV: coefficient of variation, EDTA: ethylenediaminetetraacetic acid, hrs: hours, RT: room temperature, T: time point.

**Figure A1:**
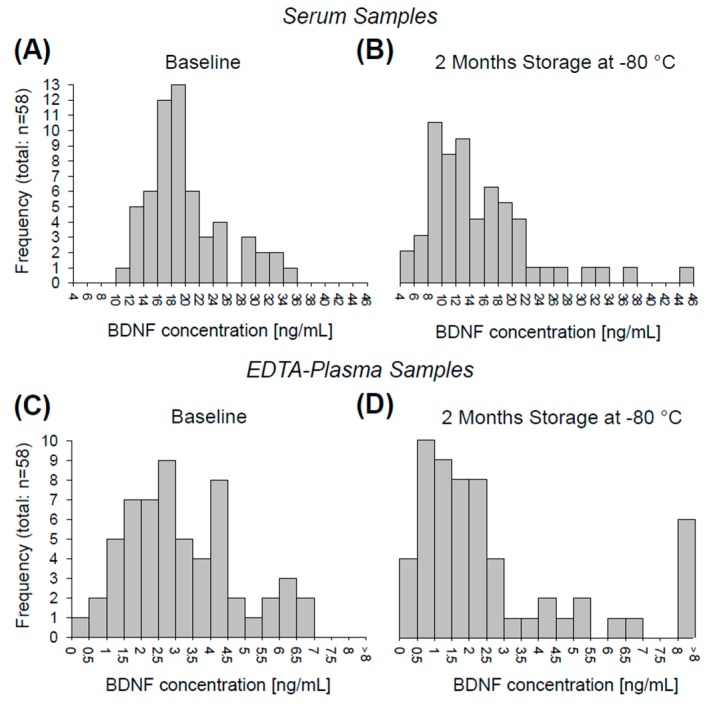
Histograms for BDNF concentrations in serum and EDTA-plasma at “Baseline” (T1) (**A**,**C**) and “2 months storage at −80 °C” (T4) (**B**,**D**). BDNF: brain-derived neurotrophic factor, EDTA: ethylenediaminetetraacetic acid.

**Figure A2:**
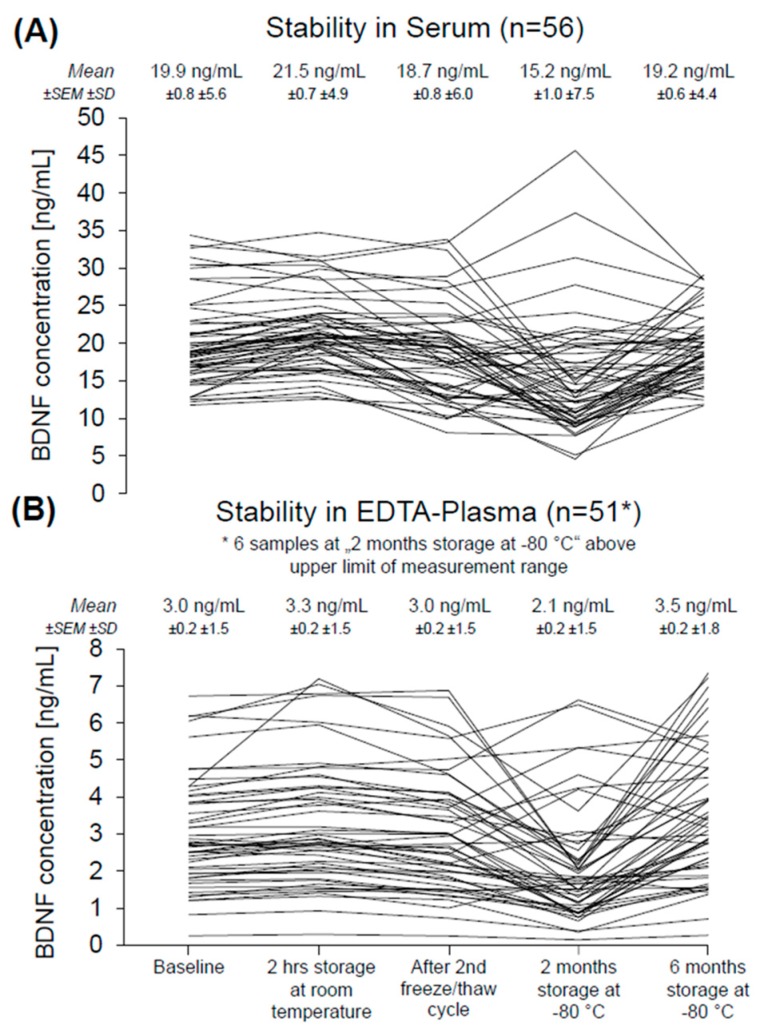
Individual changes in serum (**A)** and EDTA-plasma (**B**) BDNF from “Baseline” (T1) to “6 months storage at −80 °C” (T5). BDNF: brain-derived neurotrophic factor, EDTA: ethylenediaminetetraacetic acid, hrs: hours.

## Abbreviations

BDNF	Brain-derived neurotrophic factor
BMI	Body mass index
CV	Coefficient of variation
EDTA	Ethylenediaminetetraacetic acid
ELISA	Enzyme-linked immunosorbent assay
hrs	Hours
min	Minutes
rpm	Rounds per minute
RT	Room temperature
SD	Standard deviation
SEM	Standard error of the mean

## Appendix A

### Disscussion of Measurement Error at “2 Months Storage at −80 °C” (T4)

Results of the measurements “2 months storage at −80 °C” (T4) showed very large variations, and the coefficients of variation were 50 and 72% for the serum and EDTA-plasma samples, respectively. Furthermore, individual values differed greatly from the results obtained from the previous (“After 2nd freeze/thaw cycle”; T3) and following (“6 months storage at −80 °C”; T5) analysis of the same sample (Figure A2). Here, we present a detailed further investigation of the data and explain why we are confident that a measurement error had occurred, and why we excluded the “2 months storage at −80 °C” (T4) data from further interpretation of the data in the manuscript.

A first reason why we think that a measurement error occurred is that—in contrast to all other time points—here the results were non-normally distributed (Figure A1). Six values were above the upper limit of the measurement range. The error that occurred did not seem to be a systematic error which would have led to systematically increased or decreased values. Although mean values of serum and plasma samples decreased at this measurement, individual values changed in both directions (Figure A2). This is also reflected by the increase of standard deviation (SD) and standard error of the mean (SEM) at this measurement (for serum, increase of SD from “Baseline” (T1) to “2 months storage at −80 °C” (T4) from 5.6 to 7.8, SEM from 0.7 to 1.0; for plasma a calculation of SD and SEM was not possible because six values were above the upper limit of detection). We could not detect any pattern which explained the direction of alterations of single values. Furthermore, the positive correlation between serum and EDTA-plasma BDNF concentrations disappeared (Figure A3). The positive correlation of serum and plasma BDNF values, which we observed in the measurements at all other time points was not present at the time point “2 months storage at −80 °C” (T4) (Figure 4, Figure A3). The strongest argument for a measurement error is that the same sample was analyzed again four months later (“6 months storage at −80 °C”; T5) and similar mean BDNF values, a similar distribution, and similar serum-plasma correlations as compared to time points T1, T2, and T3 were obtained (Figure 2, Figure 3 and Figure 4, Figure A2 and Figure A3). Therefore, we conclude that the results at T4 are likely caused by a measurement error either due to a human factor or a problem with the ELISA kit or other analysis materials (e.g., buffers) on the day of analysis.

In summary, the data indicate that the measurements at T4 are not reliable. Unfortunately, we cannot assess retrospectively which laboratory procedures might have influenced the results. We decided not to interpret these data. Finally, this result underlines the necessity to interpret BDNF measurements with caution.
